# An Accurate In Vitro Model of the *E. coli* Envelope

**DOI:** 10.1002/anie.201504287

**Published:** 2015-09-01

**Authors:** Luke A Clifton, Stephen A Holt, Arwel V Hughes, Emma L Daulton, Wanatchaporn Arunmanee, Frank Heinrich, Syma Khalid, Damien Jefferies, Timothy R Charlton, John R P Webster, Christian J Kinane, Jeremy H Lakey

**Affiliations:** Institute for Cell and Molecular Biosciences, Newcastle University Framlington Place, Newcastle upon Tyne, NE2 4HH (UK) E-mail: jeremy.lakey@ncl.ac.uk; ISIS Pulsed Neutron and Muon Source, Science and Technology Facilities Council, Rutherford Appleton Laboratory Harwell Oxford Campus, Didcot, Oxfordshire, OX11 OQX (UK); Bragg Institute, Australian Nuclear Science and Technology Organisation Locked Bag 2001, Kirrawee DC, NSW 2232 (Australia); Department of Physics, Carnegie Mellon University 5000 Forbes Ave. Pittsburgh, PA 15213 (USA) National Institute of Standards and Technology Center for Neutron Research Gaithersburg, MD 20899 (USA); School of Chemistry, University of Southampton Southampton SO17 1BJ (UK)

**Keywords:** antibiotics, drug discovery, Gram-negative bacteria, membranes, structure–activity relationships

## Abstract

Gram-negative bacteria are an increasingly serious source of antibiotic-resistant infections, partly owing to their characteristic protective envelope. This complex, 20 nm thick barrier includes a highly impermeable, asymmetric bilayer outer membrane (OM), which plays a pivotal role in resisting antibacterial chemotherapy. Nevertheless, the OM molecular structure and its dynamics are poorly understood because the structure is difficult to recreate or study in vitro. The successful formation and characterization of a fully asymmetric model envelope using Langmuir–Blodgett and Langmuir–Schaefer methods is now reported. Neutron reflectivity and isotopic labeling confirmed the expected structure and asymmetry and showed that experiments with antibacterial proteins reproduced published in vivo behavior. By closely recreating natural OM behavior, this model provides a much needed robust system for antibiotic development.

Gram-negative bacteria, such as *Escherichia coli*, are increasingly displaying antibiotic resistance,[1] partly because they possess an outer membrane (OM) that forms a highly selective filter around the target cell.[2] The OM structure is unique in biology (Figure 1). Most biological membranes are lipid bilayers with partial asymmetry in lipid content between the two layers. By contrast, the OM asymmetry is profound, creating a uniquely impermeable layer. The outer leaflet is composed of lipopolysaccharide (LPS) molecules that comprise hydrophobic lipid A attached to phosphorylated sugar chains of various lengths. Divalent cations cross-link anionic LPS, which leads to the formation of a tight membrane and two hurdles for the incoming molecules to overcome. Hydrated saccharide chains prevent the ingress of hydrophobic or surface-active molecules, whilst the inner hydrophobic bilayer repels hydrophilic substances. Selective uptake through integral outer membrane proteins (OMPs) ensures that the cell receives the nutrients that it needs. These properties limit the toxicity of all antimicrobials whereas OMP mutations that further reduce OM permeability have been found in antibiotic-resistant cells.[2] Understanding the structure and function of the OM is therefore vital for human health[3] but it is challenging because bacterial cells are very small and the creation of accurate model systems is technically challenging.[4] As a result, although we have a clear knowledge of the chemical composition of the OM, our understanding of its physical and dynamic properties lags far behind our knowledge of other biological membranes.[5]

**Figure 1:**
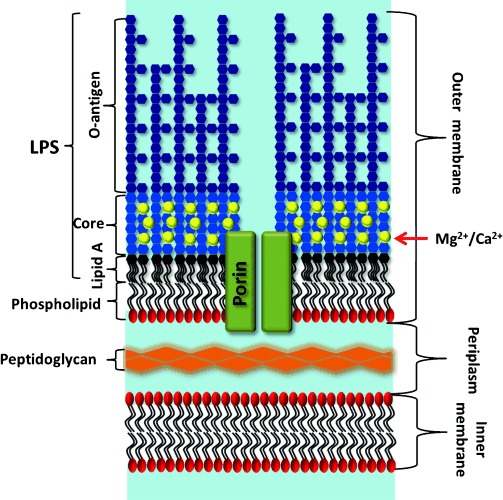
Schematic representation of the Gram-negative bacterial envelope, including the outer membrane (OM) with long “smooth” LPSs, core-associated divalent cations, integral membrane proteins (in this case, a channel-forming porin such as OmpF), and the inner phospholipid layer. The periplasm and inner membrane contain many proteins (not shown). Not to scale; the porins are approximately 5–6 nm high, the periplasm about 14 nm, and the inner membrane approximately 4 nm.

Herein, we describe a method to create asymmetric membrane models of the *E. coli* OM and reveal the unique ability of neutron reflection (NR) to confirm the asymmetry of the membrane models. The membrane “floats” on a water layer above a phosphatidylcholine self-assembled monolayer (SAM) on a smooth gold surface.[6] Although much thinner than the bacterial periplasm (ca. 145 Å),[7] which is stabilized by peptidoglycans and other polymers, the approximately 15 Å thick water layer combined with lipid asymmetry renders this floating supported bilayer (FSB) a practical, accurate, and useful synthetic OM model. The flat gold layer is adhered to the silicon substrate through an intervening permalloy layer enabling us to use magnetic-contrast NR to confidently analyze this many-layered structure.[6, 8]

First, the gold surface was coated with a SAM of ω-thiolipids.[6] The OM model was formed as an asymmetric FSB by sequential Langmuir–Blodgett and Langmuir–Schaefer deposition of a deuterium-labeled phospholipid (d-DPPC) followed by unlabeled LPS (Figure 2). The fitted data, from a series of separate FSBs, revealed neutron scattering length density (nSLD) profiles (see the Supporting Information, Figures S3–S6 and Tables S1–S4) that are consistent with asymmetric FSB on top of ω-thiolipid-coated substrates.[6, 8a] The FSB consisted of a rough mutant LPS (Ra chemotype) outer leaflet and a d-DPPC inner leaflet, which floated 12–17 Å above the choline head groups of the ω-thiolipid SAM[9] (Figure 3). These profiles were fitted to a model of the FSB that consisted of a thin inner (d-DPPC) head-group layer, inner and outer lipid-tail regions (14–18 Å), and a thick outer head-group region (28–30 Å) corresponding to the LPS core oligosaccharide (Tables S3–S6). The NR data analysis revealed FSBs coverages of >90 % (Table S2) and asymmetries ranging from 69:28 and 23:75 (LPS/PC) in the outer and inner leaflets, respectively, in the worst bilayer to 79:11 and 8:82 in the best. The thickness/nSLD profiles recorded for these bilayers were consistent with previous studies[9a, 10] and with a model generated from atomistic molecular dynamics simulations (Figure 4 A).[11] The stability of the floating model OM was examined against time and under different solution conditions. Sample 1 was analyzed over 72 h and showed no changes. The bilayers were also studied at two Ca^2+^ concentrations, 5 mm and 20 μm, and no differences in structure were observed.

**Figure 2:**
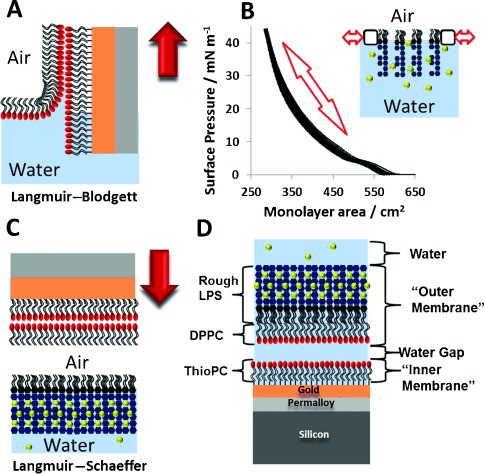
Fabrication of floating asymmetric OM models. A) Langmuir–Blodgett deposition of the DPPC layer on the SAM. B) Repeated pressure–area curves of the Ra-LPS monolayer at the air–water interface to confirm stability. C) Langmuir–Schaefer deposition of the Ra-LPS layer. D) Structure of the complete OM model (inverted to enable direct comparison with Figure 1). Red arrows indicate the direction of movement of the substrate; calcium ions are shown as yellow spheres.

**Figure 3:**
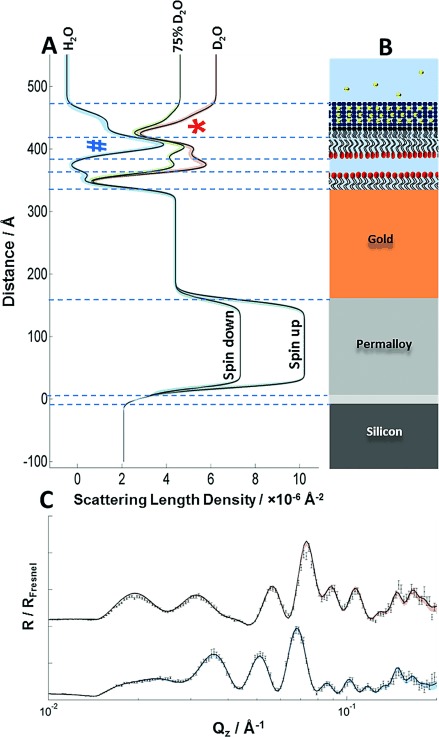
A) nSLD profiles of the OM model in different solution isotopic (H_2_O and D_2_O) contrasts and a schematic representation of the OM model. The permalloy layer provides a separate nSLD for each neutron spin. The strong peaks of the deuterated DPPC tails versus H_2_O (blue #) and the non-deuterated LPS tails versus D_2_O (red *), which confirm asymmetry, are evident. C) Original data points (black) from two spin polarizations with fitted lines that correspond to the nSLD profiles in (A). The two data sets relate to samples examined in a D_2_O buffer solution using neutrons in a spin-up configuration (red) and in a H_2_O buffer solution using spin-down neutrons (blue). Colored shading indicates the 95 % confidence limits of the fitted model, see the Supporting Information for details.

**Figure 4:**
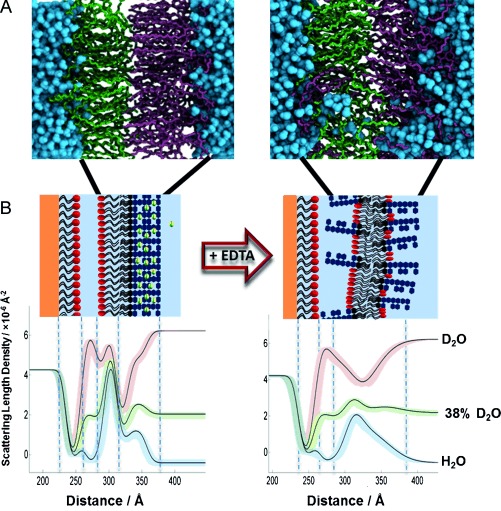
A) MD simulation of an LPS–PC asymmetric bilayer after 500 ns with Ca^2+^ (left) and 200 ns after replacement of the Ca^2+^ ions by twice the amount of Na^+^ ions (right). B) nSLD profiles obtained by fitting the neutron reflectivity data of an asymmetric DPPC/Ra-LPS system in the presence of 5 mm Ca^2+^ (left) and after calcium sequestration by EDTA (right). Schematic representations of the structures that these profiles describe are also shown, these were determined through interpretation of the fitting parameters and the resulting SLD profiles using the known scattering length densities of the bilayer components and the aqueous solutions (see the Supporting Information). Note the reduced packing/asymmetry and increased roughness.

The usefulness of this model for antibiotic development hangs upon its relevance to the natural OM. We thus tested the response of the model to either divalent-cation removal or antimicrobial proteins. Their interactions with the OM in vivo and in vitro are well known,[12] enabling direct comparisons with our model.

In vivo, divalent-cation removal by EDTA treatment of Gram-negative bacteria removes the LPS from the OM, leading to the appearance of phospholipids in the outer leaflet[12c] (which is likely due to mixing across the OM).[10] The OM model was examined in 5 mm Ca^2+^ and 3 mm EDTA solutions. Upon the sequestration of Ca^2+^ by EDTA, the bilayer asymmetry was reduced in both the inner and outer leaflets by approximately 20 % (see Figure 4 B, Figures S6, S7, and Tables S5, S6). Recently, we obtained a similar result using silicon-supported asymmetric OM mimics.[10] In the MD simulation, we imitated EDTA addition by replacing the divalent cations with twice the number of monovalent ions, keeping the whole system electroneutral. After 200 ns, the asymmetry had clearly already broken down, and the roughness of the bilayer had increased, which is in agreement with the experimental observations (Figure 4 A).

Humans produce antimicrobial proteins, including lysozyme and lactoferrin, as part of their innate immune system. Lactoferrin is cationic (pI 8.0–8.5) and acts directly upon the OM through electrostatic interactions with the anionic core oligosaccharide region.[12b] It has been suggested that it disrupts the divalent-cation bridges between neighboring LPS molecules, causing their release into the bulk solution. On the other hand, the cationic enzyme lysozyme (pI 11) is much less active against Gram-negative than against Gram-positive bacteria as it cannot easily pass through the OM to digest periplasmic peptidoglycan.[12a]

As the nSLD profiles of proteins are different from those of LPS and d-DPPC, we can define the protein layer by NR. The interaction of lactoferrin (40 μg mL^−1^) with the OM model reduced membrane coverage by 12 % and the bilayer leaflet asymmetry by approximately 30 %. The 90 Å increase in the thickness of the LPS core region (see Figure 5 A and Figures S5, S8 and Tables S5, S8) agrees with the suggested electrostatic binding of lactoferrin to the core oligosaccharide. It implies that the protein is bound to the bilayer with its major axis parallel to the membrane normal, as the prolate protein is approximately 90 Å in length along its longest axis.[13] These data provide the first structural picture of lactoferrin disrupting the OM. In the laboratory, lysozyme is often combined with EDTA to remove the outer membrane and the peptidoglycans from Gram-negative bacteria. Having already measured the large effect of EDTA, we investigated the interaction of lysozyme alone with an intact FSB in 20 μm Ca^2+^ solution. The thickness of the core oligosaccharide region increased by 20 Å, suggesting that this cationic protein has bound electrostatically to the anionic region of the outer leaflet (Figure 5 B, Figures S3, S9, Tables S3, S9). There was no loss of FSB coverage, but the bilayer roughness increased from 9.31 (range 8.35,10.0) to 12.88 (7.36,18.22) Å. Fitting of the data also suggested a minor decrease in asymmetry, albeit within error limits. These results agree with the previously observed differences[12a] in the abilities of each protein to disrupt the OM.

**Figure 5:**
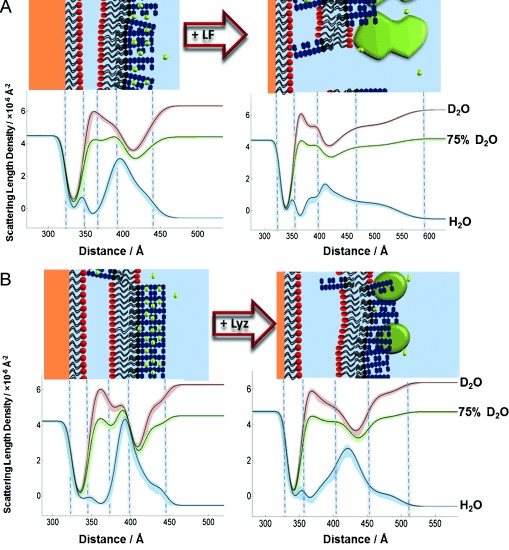
A, B) nSLD profiles and schematic representations of the OM model before and after protein addition: A) lactoferrin (LF; 40 μg mL^−1^), B) lysozyme (Lyz; 200 μg mL^−1^) in 20 mm HEPES buffer (pH/D 7.2, 20 μm CaCl_2_; see the Supporting Information for further details).

In conclusion, we have presented models of the OM that are asymmetric, rest on a water-filled layer, and contain rough bacterial lipopolysaccharides. These bilayer models are not only amenable to structural characterization by neutron reflection, but can, in principle, be studied by techniques such as surface plasmon resonance, infrared spectroscopy, atomic force microscopy, and FRET. The gold surface presents problems related to fluorescence quenching and opacity, which can be overcome by the use of PC-modified silicon substrates.[14] The models will enable studies of the interactions of antibacterial molecules, including small-molecule antibiotics,[2] polymyxins/colistins, and colicins,[15] with the OM surface under conditions that are close to, but much more tractable than, those found in vivo. Intact OMPs can be incorporated during the Langmuir–Schaeffer step, and we are currently enhancing the nSLD contrast of the OMPs to define their structure in the FSB.[16] Finally, we are developing methods to incorporate smooth lipopolysaccharides.

## Supporting Information

As a service to our authors and readers, this journal provides supporting information supplied by the authors. Such materials are peer reviewed and may be re-organized for online delivery, but are not copy-edited or typeset. Technical support issues arising from supporting information (other than missing files) should be addressed to the authors.

miscellaneous_information
