# Supplementary material for: An Accurate In Vitro Model of the E. coli Envelope
Source: Angew Chem Int Ed Engl. 2015 Sep 1;54(41):11952–5. doi: 10.1002/anie.201504287 (PMC4600229; doi:10.1002/anie.201504287)
Supplement: Supplementary file 1 — miscellaneous_information [file anie0054-11952-sd1.pdf]

Angewandte Chemie
Eine Zeitschrift der Gesellschaft Deutscher Chemiker

Supporting Information

An Accurate In Vitro Model of the *E. coli* Envelope

*Luke A. Clifton, Stephen A. Holt, Arwel V. Hughes, Emma L. Daulton, Wanatchaporn Arunmanee, Frank Heinrich, Syma Khalid, Damien Jefferies, Timothy R. Charlton, John R. P. Webster, Christian J. Kinane, and Jeremy H. Lakey**

anie_201504287_sm_miscellaneous_information.pdf

Supplementary Information

Detailed Methods

Methods

Ra mutant rough strain LPS (Ra-LPS chemotype) from EH100 *E. coli*, hen egg lysozyme and Bovine colostrum lactoferrin were obtained from Sigma-Aldrich (Dorset, UK). ω-thiolipid (1-oleoyl-2-(16-thiopalmitoyl)-sn-glycero-3-phosphocholine) and tail deuterated DPPC (d-DPPC, 1,2-dipalmitoyl(d62)-sn-glycero-3-phosphocholine) were obtained from Avanti polar lipids (Alabaster, Al, USA). All phospholipid, LPS and protein samples were used without further purification. All other chemicals were sourced from Sigma-Aldrich. The formation of the thiolipid monolayer on Permalloy/Gold coated Silicon Crystals was performed as in [1]

Gram Negative Bacterial Outer Membrane Model Deposition

Deposition of the model outer membrane models on the ω-thiolipid self-assembled monolayer (SAM) coated gold surfaces used a custom built Langmuir-Blodgett trough (Nima Technology, Coventry, UK) [1-3]. The trough was cleaned, filled with 5 mM $CaCl_2$ solution and cooled to ~10ºC. The air-liquid interface was aspirated until clean and a ω-thiolipid SAM coated silicon block was submerged in the trough.

The OM models were deposited onto the SAM coated gold surfaces in two stages; firstly the inner phospholipid leaflet of the bilayer was deposited *via* Langmuir-Blodgett (LB) deposition of a DPPC monolayer followed by deposition of the LPS outer leaflet *via* Langmuir-Schaefer (LS) transfer of a RaLPS monolayer on the crystal surface (Figure 2A). For the Langmuir-Blodgett deposition of the inner bilayer leaflet, d-DPPC was deposited from a 2 mg/ml solution in chloroform onto the air/liquid interface. Three compression and relaxation cycles of the interfacial monolayer (with a maximum pressure of 35 mN m^{-1}) were undertaken before the monolayer was compressed to 35 mN m^{-1} and the submerged ω-thiolipid SAM/gold/Permalloy coated silicon crystal was lifted through the monolayer at a speed of 4 mm/minute whilst the monolayer surface pressure was held constant (Figure 2A and Supplementary Figure 1).

The LB trough was then cleaned and refilled with 5 mM $CaCl_2$ at ~10ºC. RaLPS was then deposited onto the air/liquid interface from a 2 mg/ml suspension in chloroform: methanol: water solution (60: 39: 1 *v/v*) and, as with the DPPC monolayer, pressure cycled three times before being held at a pressure of 35 mN m^{-1}(Figure 2B). The d-DPPC/ω-thiolipid SAM coated substrate was then placed in a holder above the air/liquid interface (Figure 2C and Supplementary Figure 1B). The polished face of the silicon crystal was adjusted using a purpose built levelling device to make crystal face parallel to the water surface. The silicon crystal (and LB film) was then dipped through the interface at a constant speed of 4 mm/min and lowered into a purpose built sample cell in the well of the trough (Figure 2C Supplementary Figure 2).

NR measurements of the OM models were undertaken on the POLREF and CRISP [4] white beam reflectometers at the Rutherford Appleton Laboratory (Oxfordshire, UK), both of which are able to operate in a polarized mode (which was used for the analysis of the majority of samples discussed here) [5]. NR measures the neutron reflection as a function of the angle and/or wavelength (λ) of the beam relative to the sample [6, 7]. The reflected intensity was measured as a function of the momentum transfer, Q_z ($Q_z = (4\pi \sin \theta)/\lambda$ where λ is wavelength and θ is the incident angle). The white beam instruments are able probe a wide area of Q_z space at a single angle of reflection due to the use of a broad neutron spectrum. Therefore, to obtain reflectivity data across a Q_z range of ~0.01 to 0.3 glancing angles of 0.35º, 0.8º and 1.5º were used in CRISP, which has a λ range of 0.5 to 6.5 Å [6], and 0.5°, 1.0° and 2.3° for POLREF (λ = 2 to 12 Å).

The samples are placed in a magnetic field [8, 9]. The two spin orientations result in two distinct nSLD for the permalloy layer but unchanged nSLD for the rest of the sample. The bilayer structure was analysed in three solution isotopic contrasts (i) 100% D_2O, (ii) 75% D_2O (which has the same nSLD as gold and thus called Au matched water, AuMW; in one case silicon matched water, SiMW; 38% D_2O, was used instead of AuMW).) or (iii) 100% H_2O. SiMW and AuMW are used in order to simplify the layer structure by effectively making the biological layer (AuMW) or biological layer plus gold (SiMW) stand out from a completely blank background on either side.

For the interaction of antimicrobial proteins with the model OM lyophilized protein samples were dissolved in 20 mM HEPES pH/D 7.2 20 µM $CaCl_2$ buffer solutions in H_2O and D_2O which were then flowed into the sample cells in the appropriate H/D mix required for the isotopic contrast under examination.

Neutron reflectivity data were analysed using the in-house software, RasCal (A. Hughes, , Rutherford Appleton Laboratory [10]) which fits layer models describing the interfacial structure calculated using the recursive Parratt formalism [11] to the experimental reflectivity data. In this approach the interface is described as a series of slabs,

each of which is characterised by its nSLD, thickness and roughness. The reflectivity for the model starting point is then calculated and compared with the experimental data.

For the SAM layers, the models were parameterised in terms of partial lipid volumes[12] as described previously[1]. The floating bilayers were described as four layers (inner headgroup, inner tails, outer tail, outer headgroup) each layer defined by nSLD (See Supplementary Table 1), thickness and roughness [2, 13]. The samples were examined under three solution contrasts, with each contrast examined with two polarizations of the neutron beam (spin up and down) to yield six reflectivity profiles for each structure examined (Supplementary Figs and Tables 2-4 & 7-8). The six reflectivity profiles were constrained to fit to a single profile of layer thickness and roughness across the interface but the data fits from each isotopic contrast were allowed to vary in the nSLD of each hydrated layer in order to account for water/labile hydrogen content of the sample and its volume fraction[13]. In one case (sample 4), a non-polarized neutron beam was used. In this case three datasets (from three differing solution isotopic contrast conditions) were fitted instead of six (Supplementary Figs and Tables 5 & 6).

For bilayer only samples (i.e. without adsorbed protein either in the presence of Ca^{2+} or EDTA) the parameter fit values and the scattering length density profiles these describe were used to determine the bilayer coverage (i.e. volume fraction of bilayer defects across the surface [2, 13]). The lipid asymmetry was determined from the nSLD of the tail regions of the bilayer using previously described linear equations [2, 13].

For antimicrobial protein interaction studies, the binding and membrane disruptive effects of lactoferrin and lysozyme were determined by comparing the nSLD profiles of the floating bilayer region of the sample before and after protein introduction. Protein binding to the core oligosaccharide region of the bilayer was determined by thickness changes in the bilayers' outer leaflet headgroup region. Membrane disruption was determined by a loss of bilayer tail asymmetry; due to leaflet mixing and an increase in bilayer hydration; due to the formation of water filled membrane defects.

Models were fitted to the data using a Bayesian approach [14, 15] with the log-likelihood function described in terms of the overall chi-squared [14]. Priors were either uniform or Gaussian as described in the supplementary information. In addition to the model parameters, the backgrounds, scale factors and instrument resolutions were also fitted. Marginalised posteriors were obtained using a Delayed Rejection Adaptive Metropolis algorithm [16, 17]and the best fit parameters taken as the distribution maxima, and the uncertainties obtained from the shortest 95% confidence intervals of each distribution (Table 2).

Molecular Dynamics

Full details of the previously published *in silico* asymmetric *E. coli* model outer membrane are given in [18] and, compared to the *in vitro* model, it uses shorter LPS and a more complex inner membrane phospholipid mixture. The outer leaflet of the asymmetric bilayer was composed entirely of Rd_1 LPS molecules. The inner leaflet of the membrane was composed of a mixture of PE (90%), PG (5%) and cardiolipin (5%) phospholipids. The phospholipid fatty acyl tail composition of the inner leaflet was (1-palmitoly, 2-cis-vaccenyl for PE and PG and 1-palmitoyl, 2- cis-vaccenyl, 3-pamitoly, 4- cis-vaccenyl for cardiolipin.

All simulations were performed using the GROMACS package [19-21] version 4.5.5 (GROMOS 53A6 force field [22]) and the SPC water model [23]. During the simulations the LPS, phospholipids and solvent (water plus ions) were maintained at a constant temperature above the gel (Lβ) to liquid crystal (Lα) phase transition temperatures using the Nosé-Hoover thermostat with a time constant of 0.5 ps [24, 25] A pressure of 1 bar was maintained using anisotropic pressure coupling with the Parrinello-Rahman barostat and a time constant of 5 ps [26, 27]. Electrostatic interactions were treated using the smooth particle mesh Ewald (PME) algorithm [28] with a short-range cut-off of 1.2 nm. Van der Waals interactions were truncated at 1.2 nm with a long-range dispersion correction applied to the energy and pressure. The neighbour list was updated every 5 steps during the simulations. All bonds were constrained using the LINCS algorithm [29] allowing a 2 fs time step to be applied. After 500 ns of simulation of our previously reported outer membrane model [18], we removed all divalent cations ions from the system and replaced them with twice the number of monovalent cations ions, and simulated this system for a further 200 ns.

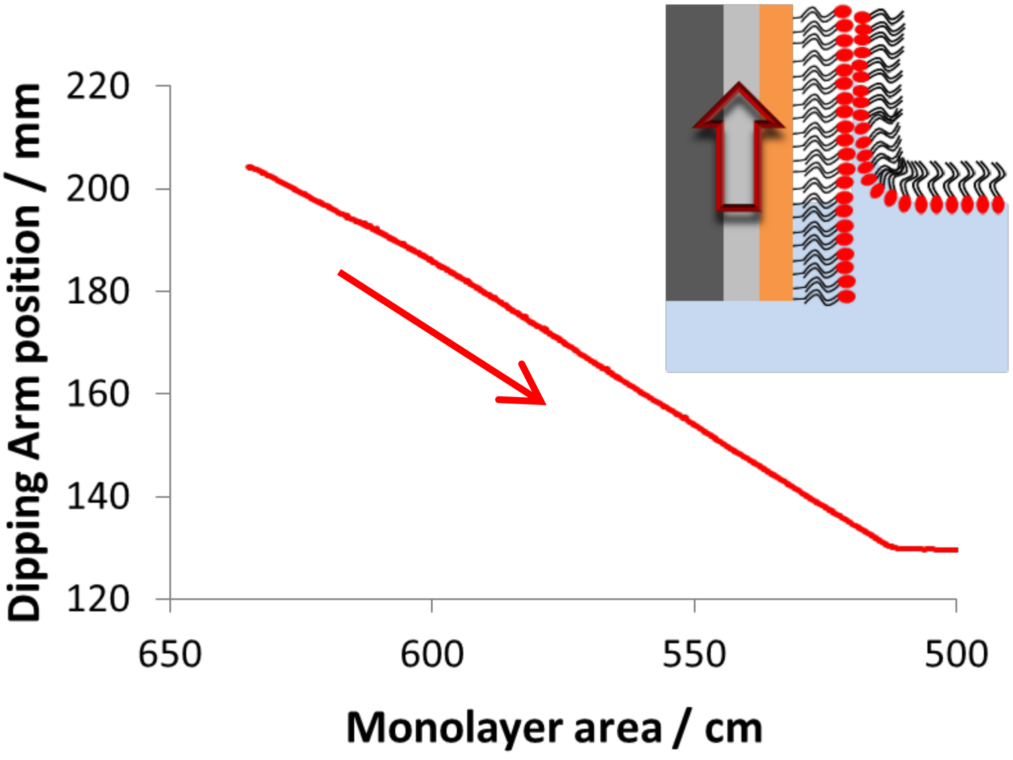

Figure S1, Dipping arm position *vs.* Monolayer area plot obtained during the Langmuir-Blodgett deposition of a tail deuterated DPPC monolayer held at 35 mN m^{-1} to the surface of a ω-thiolipid/gold/Permalloy coated silicon crystal. A diagrammatic representation of the process is shown.

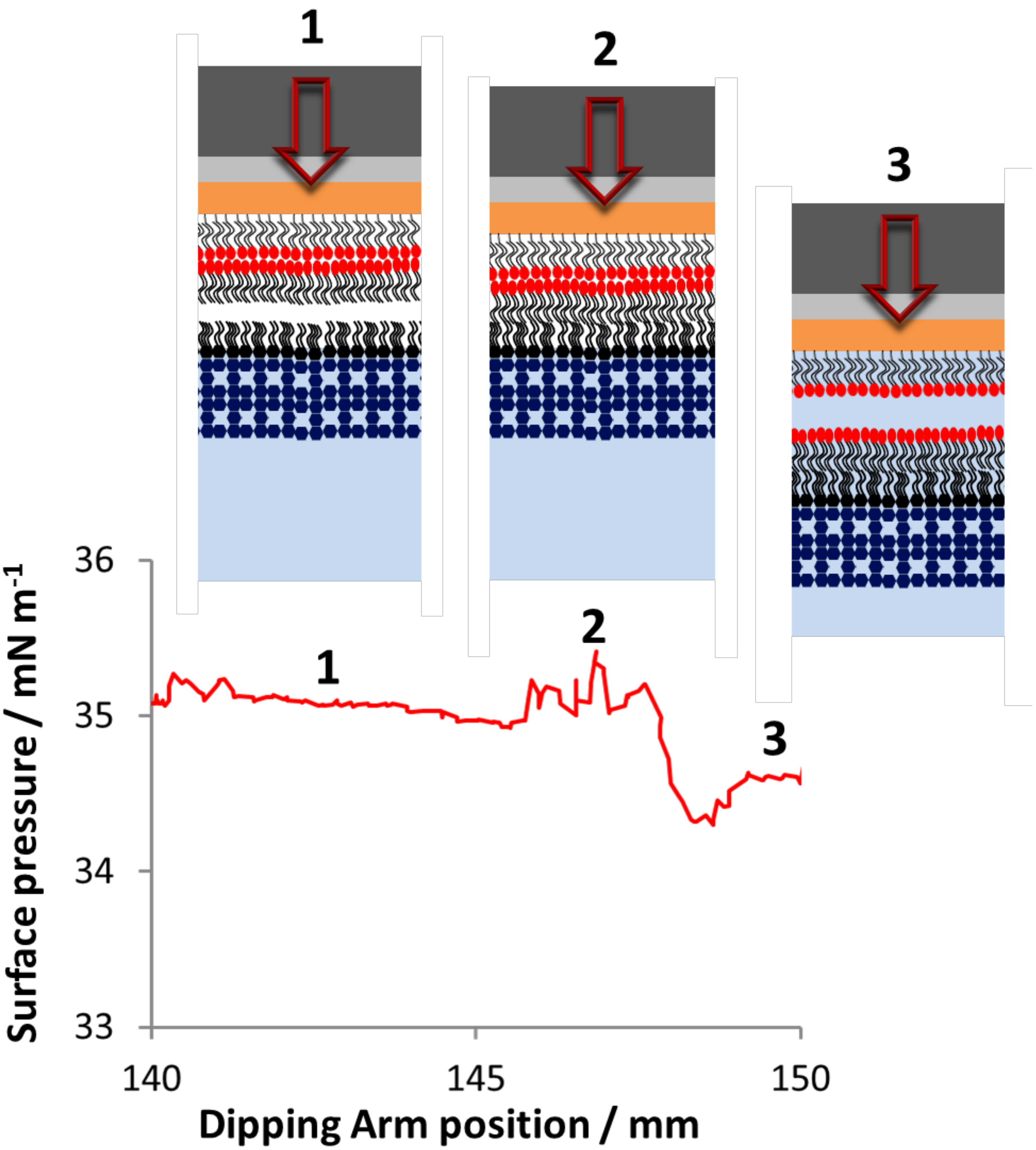

Figure S2, Surface pressure vs. dipping arm position plot obtained for the Langmuir-Schaefer deposition of a LPS monolayer held at 35 mN m-1 onto the surface of a d-DPPC/ω-thiolipid/gold/Permalloy coated silicon crystal. Diagrams of the process are shown before (1), during (2) and after (3) Langmuir-Schaefer deposition of the LPS monolayer.

Table S1, Summary of known scattering length densities of the lipid components, proteins and the solution subphases.

Lipid / Solvent	Neutron scattering length density (ρ) (10^{-6} Å^{-2})
D_2O	6.35
Gold matched water (AuMW), 75% D_2O	4.66
Silicon matched water (SiMW), 38% D_2O	2.07
H_2O	-0.56
Silicon	2.07
Silicon oxide (SiO_2)	3.41
Gold	4.66
DPPC head group	1.98
h-DPPC tails	-0.37
d-DPPC tails	7.45
LPS tails	-0.37
LPS core oligosaccharide in D_2O	4.28
LPS core oligosaccharide in H_2O	2.01
Lysozyme in D_2O	3.45
Lysozyme in H_2O	1.98
Lactoferrin in D_2O	3.15
Lactoferrin in H_2O	1.87

Table S2, Summary of bilayer asymmetry and coverage for asymmetric DPPC/RaLPS bilayer deposited on ω-thiolipid/gold/Permalloy coated silicon crystals. Values have been rounded to whole numbers, brackets display 95 % confidence limits).

Sample	Thiolipid SAM coverage	DPPC/RaLPS bilayer coverage	Inner leaflet lipid contents	Outer leaflet lipid contents
1	98% (96,100)	95 % (91,99)	81% (73, 90) DPPC<br>13% (5, 22) LPS	28% (13, 29) DPPC<br>76% (71, 82) LPS
2	97% (95, 100)	98% (95, 99)	75% (70, 83) DPPC<br>23% (15, 28) LPS	28% (25, 32) DPPC<br>69% (66, 73) LPS
3	93% (90, 96)	91% (86, 96)	76% (70, 83) DPPC<br>15% (8, 21) LPS	21% (14, 27) DPPC<br>70% (64, 77) LPS
4	97% (95,100)	90% (86, 94)	82% (73,100) DPPC<br>8% (0,17) LPS	11% (2, 17) DPPC<br>79% (73, 88) LPS

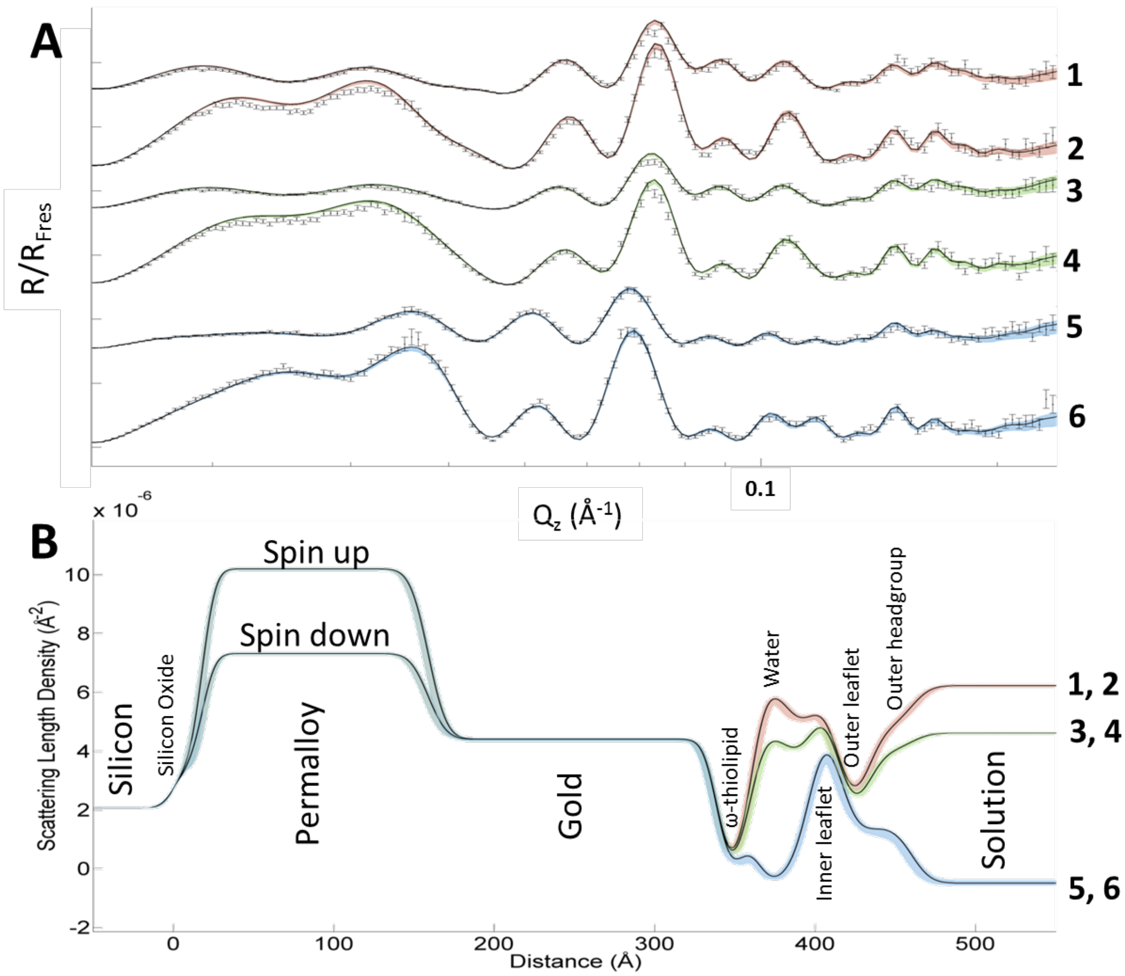

Figure S3, Neutron reflectometry profile and model data fits (A) and the scattering length density profiles these fits describe (B) for sample 1, an asymmetric d-DPPC (inner leaflet) : RaLPS (outer leaflet) bilayer deposited onto a ω-thiolipid SAM/gold/Permalloy coated silicon crystal in the presence of 20 mM HEPES pH/D 7.2 buffer with 5 mM $CaCl_2$. The six simultaneously fitted isotopic and magnetic contrasts are from the sample measured in a spin down (1) and spin up (2) beam (with respect to the magnetization of the Permalloy layer) in a D_2O containing buffer subphase, spin down (3) and spin up (4) beam configuration in gold matched water (AuMW, 75% D_2O) containing subphase and spin down (5) and spin up (6) beam configuration in a H_2O containing buffer subphase. Coloured shaded areas indicate the 95% confidence limits.

Table S3. Best-fit values (maxima of the parameter probability histograms from the Bayesian analysis) obtained from the fitting of asymmetric d-DPPC/RaLPS bilayer sample 1. The bracketed ranges are the shortest 95% confidence intervals.

Parameter	*Fitted Values*	*Priors*
**Substrate Roughness (Å)**	6.54 (5.75, 7.30)	uniform (min = 0.1, max = 20)
**Oxide thickness (Å)**	17.59 (15.2, 19.50)	uniform (min = 0, max = 30)
**Permalloy thickness (Å)**	140.73 (140.38, 141.12)	uniform (min = 10, max = 200)
**Permalloy SLD spin down beam (Å^{-2})**	7.32e-06 (7.30e-06, 7.35e-06)	uniform (min = 5e-06, max = 9e-06)
**Permalloy SLD spin up beam (Å^{-2})**	10.20e-06 (10.17e-06, 10.23e-06)	uniform (min = 8e-06, max = 13e-06)
**Permalloy Roughness (Å)**	9.013 (8.60, 9.41)	uniform (min = 0.1, max = 10)
**Gold Thickness (Å)**	179.67 (179.22, 180.11)	uniform (min = 50, max = 200)
**Gold Roughness (Å)**	6.94 (6.58, 7.26)	uniform (min = 2, max = 20)
**Gold SLD (Å^{-2})**	4.41e-06 (4.37e-06, 4.45e-06)	uniform (min = 4.0e-06, max = 5e-06)
**ω-thiolipid area (Å^{2})**	49.077 (47.08, 50.81)	uniform (min = 40, max = 100)
**ω-thiolipid head coverage (%)**	89 (72, 99)	uniform (min = 0, max = 100)
**ω-thiolipid coverage (%)**	98 (96, 100)	uniform (min = 50, max = 100)
**Central water thickness (Å)**	21.63 (19.73, 23.54)	gaussian (min = 2, max = 27)
**Inner head thickness (Å)**	14.22 (12.56, 15.97)	gaussian (min = 5, max = 40)
**Inner head SLD (Å^{-2})**	1.35e-06 (6.61e-07, 2.08e-06)	gaussian (min = 0, max = 5e-06)
**Inner head hydration (%)**	60 (52, 68)	uniform (min = 0, max = 100)
**Inner tails SLD (Å^{-2})**	6.3096e-06 (5.6697e-06, 7.035e-06)	uniform (min = -4e-07, max = 8e-06)
**Inner tails thickness (Å)**	15.744 (14.055, 17.604)	gaussian (min = 7, max = 30)
**Outer tails SLD (Å^{-2})**	1.212e-06 (6.7621e-07, 1.6216e-06)	uniform (min = -4e-07, max = 8e-06)
**Outer tails thickness (Å)**	17.724 (15.58, 19.714)	gaussian (min = 10, max = 30)
**Tails coverage (%)**	0.95048 (0.91448, 0.98979)	uniform (min = 0, max = 100)
**Outer head SLD H_2O (Å^{-2})**	2.8451e-06 (1.6241e-06, 3.9293e-06)	uniform (min = 1e-06, max = 4e-06)
**Outer head SLD D_2O (Å^{-2})**	3.8112e-06 (2.9514e-06, 4.7439e-06)	uniform (min = -5e-07, max = 6e-06)
**Outer head thickness (Å)**	28.12 (26.46, 29.61)	uniform (min = 8, max = 40)

**Outer head hydration (%)**	43 (15, 59)	uniform (min = 0, max = 100)
**Bilayer roughness (Å)**	9.31 (8.35, 10)	uniform (min = 2, max = 10)

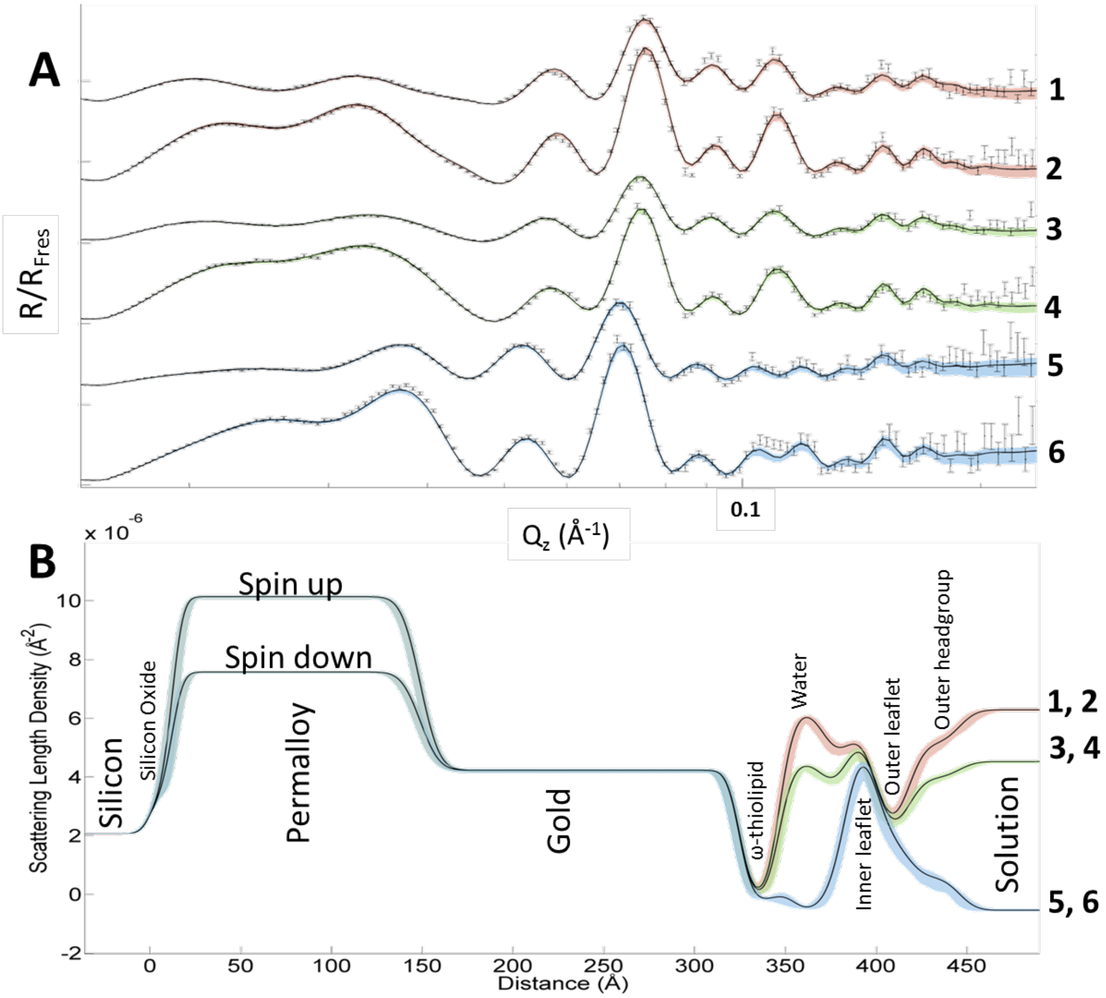

Figure S4, Neutron reflectometry profile and model data fits (A) and the scattering length density profiles these fits describe (B) for sample 2, an asymmetric d-DPPC (inner leaflet) : RaLPS (outer leaflet) bilayer deposited onto a ω-thiolipid SAM/gold/Permalloy coated silicon crystal in the presence of 20 mM HEPES pH/D 7.2 buffer with 5 mM $CaCl_2$. The six simultaneously fitted isotopic and magnetic contrasts are from the sample measured in a spin down (1) and spin up (2) beam (with respect to the magnetization of the Permalloy layer) in a D_2O containing buffer subphase, spin down (3) and spin up (4) beam configuration in gold matched water (AuMW, 75% D_2O) containing subphase and spin down (5) and spin up (6) beam configuration in a H_2O containing buffer subphase. Coloured shaded areas indicate the 95% confidence limits.

Table S4, Best-fit values (maxima of the parameter probability histograms from the Bayesian analysis) obtained from the fitting of the asymmetric d-DPPC/RaLPS bilayer sample 2. The bracketed ranges are the shortest 95% confidence intervals.

Parameter	***Fitted Values***	***Priors***
**Substrate Roughness (Å)**	4.80 (4.06, 5.80)	uniform (min = 0.1, max = 20)
**Oxide thickness (Å)**	11.71 (8.80, 13.96)	uniform (min = 0, max = 30)
**Permalloy thickness (Å)**	136.57 (136.13, 137.14)	uniform (min = 10, max = 200)
**Permalloy SLD spin down beam ($Å^{-2}$)**	7.57e-06 (7.55e-06, 7.60e-06)	uniform (min = 5e-06, max = 9e-06)
**Permalloy SLD spin up beam ($Å^{-2}$)**	10.14e-06 (10.10e-05, 10.18e-05)	uniform (min = 8e-06, max = 13e-06)
**Permalloy Roughness (Å)**	8.24 (7.72, 8.76)	uniform (min = 0.1, max = 10)
**Gold Thickness (Å)**	175.68 (175.24, 176.16)	uniform (min = 50, max = 200)
**Gold Roughness (Å)**	5.59(5.06, 6.22)	uniform (min = 2, max = 20)
**Gold SLD ($Å^{-2}$)**	4.23e-06 (4.19e-06, 4.28e-06)	uniform (min = 4.4e-06, max = 5e-06)
**ω-thiolipid area ($Å^2$)**	40.13 (40.00, 40.43)	uniform (min = 40, max = 100)
**ω-thiolipid head coverage (%)**	26 (14, 38)	uniform (min = 0, max = 100)
**ω-thiolipid coverage (%)**	98 (95, 100)	uniform (min = 50, max = 100)
**Central water thickness (Å)**	18.35 (16.24, 20.43)	gaussian (min = 2, max = 27)
**Inner head thickness (Å)**	11.032(9.30, 12.88)	gaussian (min = 5, max = 40)
**Inner head SLD ($Å^{-2}$)**	1.11e-06 (5.77e-07, 1.79e-06)	gaussian (min = 0, max = 5e-06)
**Inner head hydration (%)**	61 (49, 70)	uniform (min = 0, max = 100)
**Inner tails SLD ($Å^{-2}$)**	5.65e-06 (5.22e-06, 6.24e-06)	uniform (min = -4e-07, max = 8e-06)
**Inner tails thickness (Å)**	16.33 (14.45, 18.24)	gaussian (min = 7, max = 30)
**Outer tails SLD ($Å^{-2}$)**	1.91e-06 (1.63e-06, 2.18e-06)	uniform (min = -4e-07, max = 8e-06)
**Outer tails thickness (Å)**	17.86 (15.86, 19.77)	gaussian (min = 10, max = 30)
**Tails coverage (%)**	98 (95, 100)	uniform (min = 0, max = 100)
**Outer head SLD H_2O ($Å^{-2}$)**	2.85e-06 (1.67e-06, 3.94e-06)	uniform (min = 1e-06, max = 4e-06)
**Outer head SLD D_2O ($Å^{-2}$)**	2.82e-06 (1.55e-06, 4.03e-06)	uniform (min = -5e-07, max = 6e-06)
**Outer head thickness (Å)**	28.26 (25.71, 30.94)	uniform (min = 8, max = 40)

**Outer head hydration (%)**	67(0.53, 0.78)	uniform (min = 0, max = 100)
**Bilayer roughness (Å)**	7.40 (6.00, 8.55)	uniform (min = 2, max = 10)

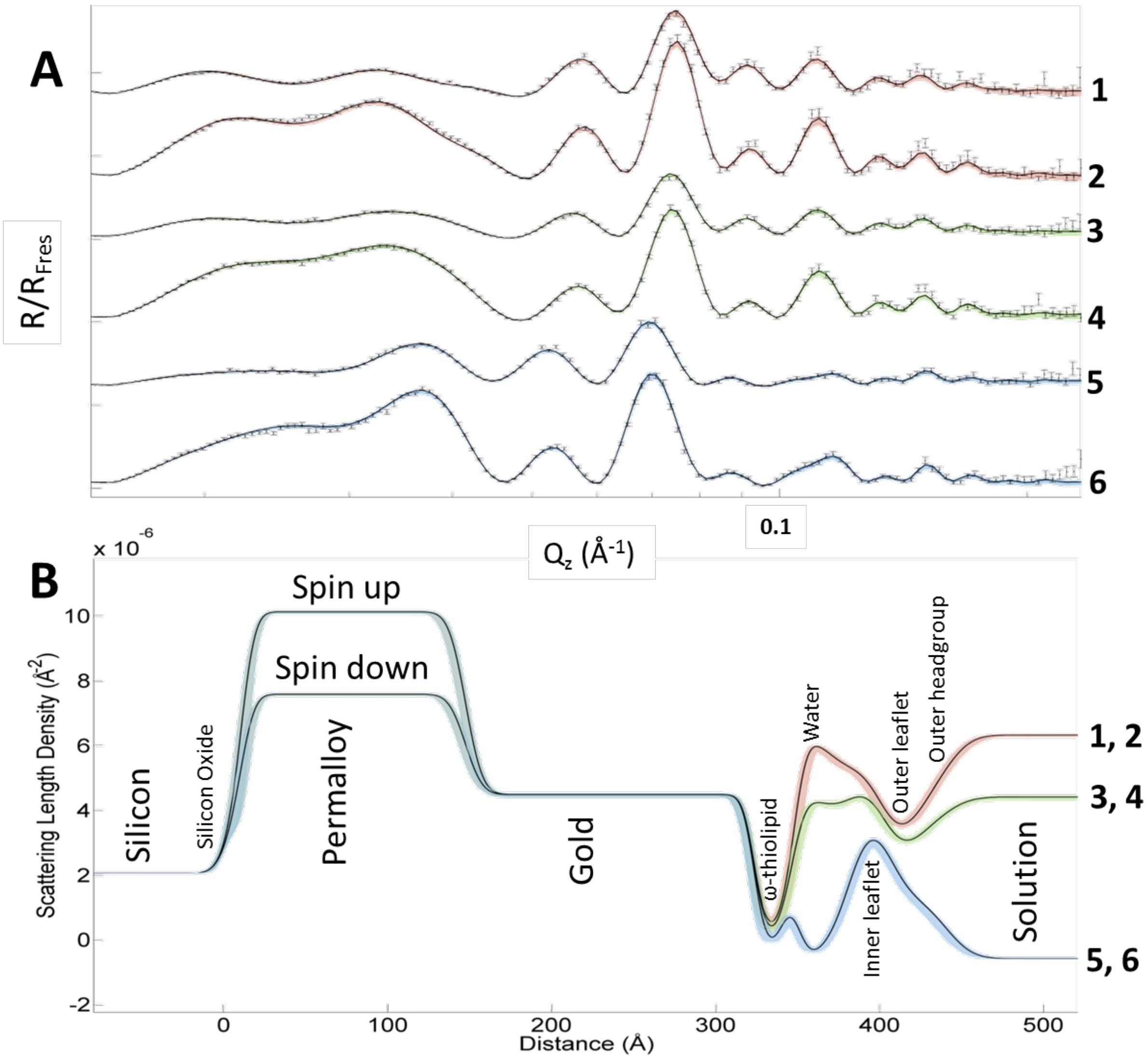

Figure S5, Neutron reflectometry profile and model data fits (A) and the scattering length density profiles these fits describe (B) for sample 3, an asymmetric d-DPPC (inner leaflet) : RaLPS (outer leaflet) bilayer deposited onto a ω-thiolipid SAM/gold/Permalloy coated silicon crystal in the presence of 20 mM HEPES pH/D 7.2 buffer with 5 mM $CaCl_2$. The six simultaneously fitted isotopic and magnetic contrasts are from the sample measured in a spin down (1) and spin up (2) beam (with respect to the magnetization of the Permalloy layer) in a D_2O containing buffer subphase, spin down (3) and spin up (4) beam configuration in gold matched water (AuMW, 75% D_2O) containing subphase and spin down (5) and spin up (6) beam configuration in a H_2O containing buffer subphase. Coloured shaded areas indicate the 95% confidence limits.

Table S5, Best-fit values (maxima of the parameter probability histograms from the Bayesian analysis) obtained from the fitting of the asymmetric d-DPPC/RaLPS bilayer sample 3. The bracketed ranges are the shortest 95% confidence intervals.

Parameter	*Fitted Values*	*Priors*
**Substrate Roughness (Å)**	6.54 (5.71, 7.33)	uniform (min = 0.1, max = 20)
**Oxide thickness (Å)**	10.54 (7.32, 13.25)	uniform (min = 0, max = 30)
**Permalloy thickness (Å)**	136.39 (135.86, 136.99)	uniform (min = 10, max = 200)
**Permalloy SLD spin down beam ($Å^{-2}$)**	7.59e-06 (7.55e-06, 7.63e-06)	uniform (min = 5e-06, max = 9e-06)
**Permalloy SLD spin up beam ($Å^{-2}$)**	10.13e-06 (10.09e-06, 10.17e-06)	uniform (min = 8e-06, max = 13e-06)
**Permalloy Roughness (Å)**	8.14 (7.71, 8.57)	uniform (min = 0.1, max = 10)
**Gold Thickness (Å)**	175.78 (175.40, 176.21)	uniform (min = 50, max = 200)
**Gold Roughness (Å)**	5.69 (5.12, 6.23)	uniform (min = 2, max = 20)
**Gold SLD ($Å^{-2}$)**	4.49e-06 (4.43e-06, 4.54e-06)	uniform (min = 4.4e-06, max = 5e-06)
**ω-thiolipid area ($Å^2$)**	45.13 (43.89, 46.28)	uniform (min = 40, max = 100)
**ω-thiolipid head coverage (%)**	96 (87, 100)	uniform (min = 0, max = 100)
**ω-thiolipid coverage (%)**	93 (90, 96)	uniform (min = 50, max = 100)
**Central water thickness (Å)**	23.87 (21.78, 26.15)	gaussian (min = 2, max = 27)
**Inner head thickness (Å)**	12.29 (10.21, 14.21)	gaussian (min = 5, max = 40)
**Inner head SLD ($Å^{-2}$)**	1.22e-06 (6.07e-07, 1.86e-06)	gaussian (min = 0, max = 5e-06)
**Inner head hydration (%)**	62 (51, 71)	uniform (min = 0, max = 100)
**Inner tails SLD ($Å^{-2}$)**	6.2e-06 (5.61e-06, 6.87e-06)	uniform (min = -4e-07, max = 8e-06)
**Inner tails thickness (Å)**	16.11 (14.24, 18.03)	gaussian (min = 7, max = 30)
**Outer tails SLD ($Å^{-2}$)**	1.44e-06 (8.22e-07, 1.94e-06)	uniform (min = -4e-07, max = 8e-06)
**Outer tails thickness (Å)**	15.62 (13.53, 17.75)	gaussian (min = 10, max = 30)
**Tails coverage (%)**	91 (86, 96)	uniform (min = 0, max = 100)
**Outer head SLD H_2O ($Å^{-2}$)**	2.65e-06 (1.33e-06, 3.90e-06)	uniform (min = 1e-06, max = 4e-06)
**Outer head SLD D_2O ($Å^{-2}$)**	2.95e-06 (1.53e-06, 4.39e-06)	uniform (min = -5e-07, max = 6e-06)
**Outer head thickness (Å)**	23.66 (21.17, 26.25)	uniform (min = 8, max = 40)

**Outer head hydration (%)**	44 (13, 63)	uniform (min = 0, max = 100)
**Bilayer roughness (Å)**	7.40 (6.00, 8.55)	uniform (min = 2, max = 10)

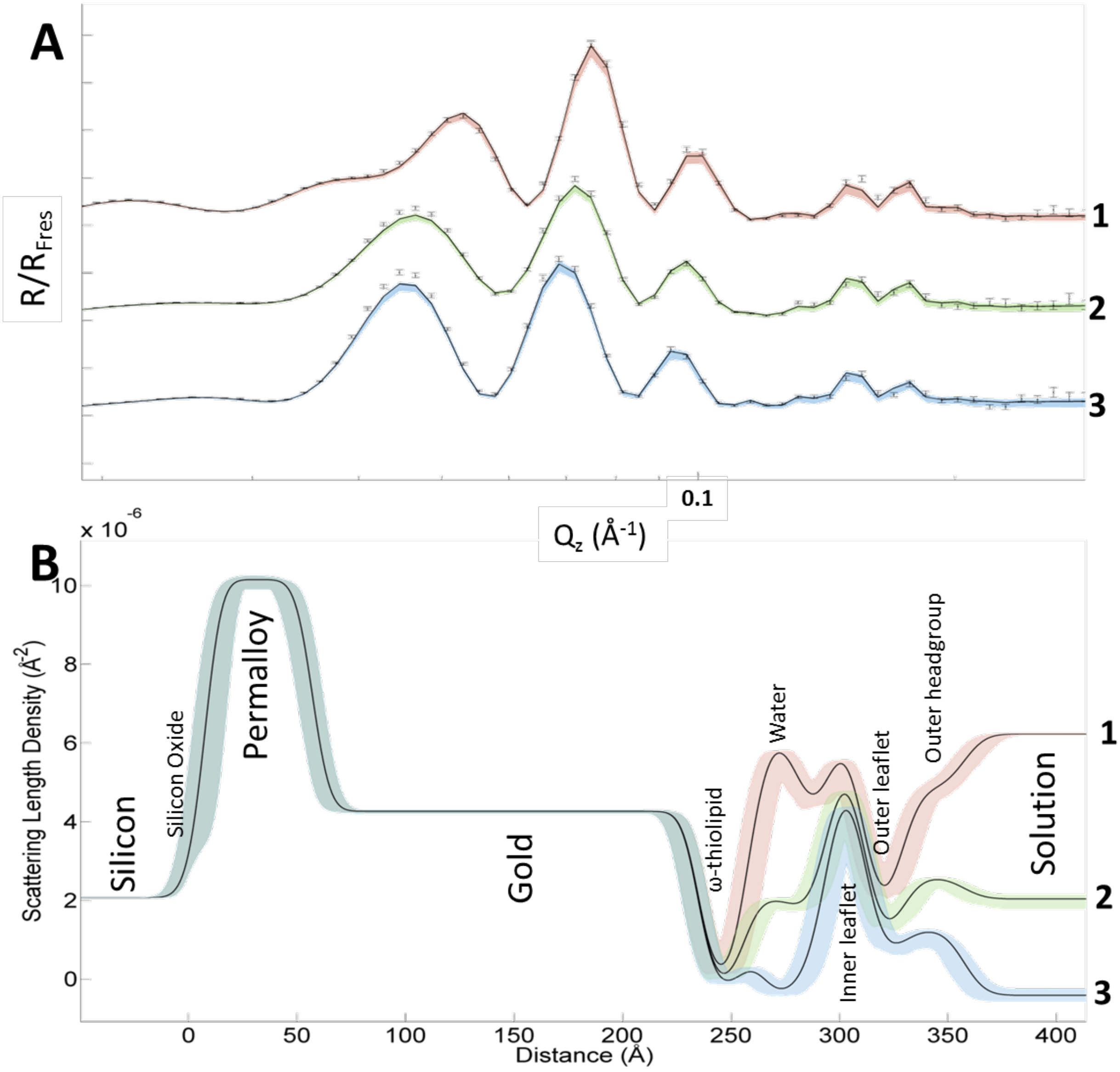

Figure S6, Neutron reflectometry profile and model data fits (A) and the scattering length density profiles these fits describe (B) for sample 4, an asymmetric d-DPPC (inner leaflet) : RaLPS (outer leaflet) bilayer deposited onto a ω-thiolipid SAM/gold/Permalloy coated silicon crystal in the presence of 20 mM HEPES pH/D 7.2 buffer with 5 mM $CaCl_2$. The three simultaneously fitted isotopic contrasts are from the sample in D_2O (1), silicon matched water (SiMW, 38% D_2O; 2) and in a H_2O (3) containing buffer subphases. Coloured shaded areas indicate the 95% confidence limits. Note how these are larger when fitting only three contrasts in the absence of two polarization sets.

Table S6, Best-fit values (maxima of the parameter probability histograms from the Bayesian analysis) obtained from the fitting of the asymmetric d-DPPC/RaLPS bilayer sample 4 in the presence of 5 mM $CaCl_2$. The bracketed ranges are the shortest 95% confidence intervals.

Parameter	***Fitted Values***	***Priors***
**Substrate Roughness (Å)**	5.76 (4.20, 7.09)	uniform (min = 0.1, max = 20)
**Oxide thickness (Å)**	7.7849 (0.6029, 13.84)	uniform (min = 0, max = 30)
**Permalloy thickness (Å)**	48.761 (47.706, 49.961)	uniform (min = 20, max = 60)
**Permalloy Roughness (Å)**	6.3213 (5.455, 7.146)	uniform (min = 0.1, max = 10)
**Permalloy SLD ($Å^{-2}$)**	10.15e-06 (10.03e-06, 10.26e-06)	uniform (min = 5e-06, max = 1.3e-05)
**Gold Thickness (Å)**	177.61 (177.03, 178.24)	uniform (min = 50, max = 200)
**Gold Roughness (Å)**	6.167 (5.44, 6.87)	uniform (min = 2, max = 20)
**Gold SLD ($Å^{-2}$)**	4.27e-06 (4.24e-06, 4.30e-06)	uniform (min = 4.4e-06, max = 5e-06)
**ω-thiolipid area ($Å^2$)**	40.17 (40.005, 40.594)	uniform (min = 40, max = 100)
**ω-thiolipid head coverage (%)**	46 (34, 59)	uniform (min = 0, max = 100)
**ω-thiolipid coverage (%)**	97 (94, 100)	uniform (min = 50, max = 100)
**Central water thickness (Å)**	17.845(15.76, 19.99)	gaussian (min = 2, max = 27)
**Inner head thickness (Å)**	12.89 (11.04, 14.92)	gaussian (min = 5, max = 40)
**Inner head SLD ($Å^{-2}$)**	0.81e-06 (0.51e-06, 1.33e-06)	gaussian (min = 0, max = 5e-06)
**Inner head hydration (%)**	50 (38, 59)	uniform (min = 0, max = 100)
**Inner tails SLD ($Å^{-2}$)**	6.76e-06 (6.02e-06, 7.66e-06)	uniform (min = -4e-07, max = 8e-06)
**Inner tails thickness (Å)**	15.98 (14.11, 17.88)	gaussian (min = 7, max = 30)
**Outer tails SLD ($Å^{-2}$)**	0.55e-06 (-0.19e-06, 1.14e-06)	uniform (min = -4e-07, max = 8e-06)
**Outer tails thickness (Å)**	16.91 (14.58, 19.22)	gaussian (min = 10, max = 30)
**Tails coverage (%)**	90 (86, 94)	uniform (min = 0, max = 100)
**Outer head SLD H_2O ($Å^{-2}$)**	2.81e-06 (1.62e-06, 3.93e-06)	uniform (min = 1e-06, max = 4e-06)
**Outer head SLD D_2O ($Å^{-2}$)**	3.57e-06 (2.52e-06, 4.59e-06)	uniform (min = -5e-07, max = 6e-06)
**Outer head thickness (Å)**	29.65 (27.35, 32.09)	uniform (min = 8, max = 40)

**Outer head hydration (%)**	49 (24, 64)	uniform (min = 0, max = 100)
**Bilayer roughness (Å)**	7.74 (6.57, 8.84)	uniform (min = 2, max = 10)

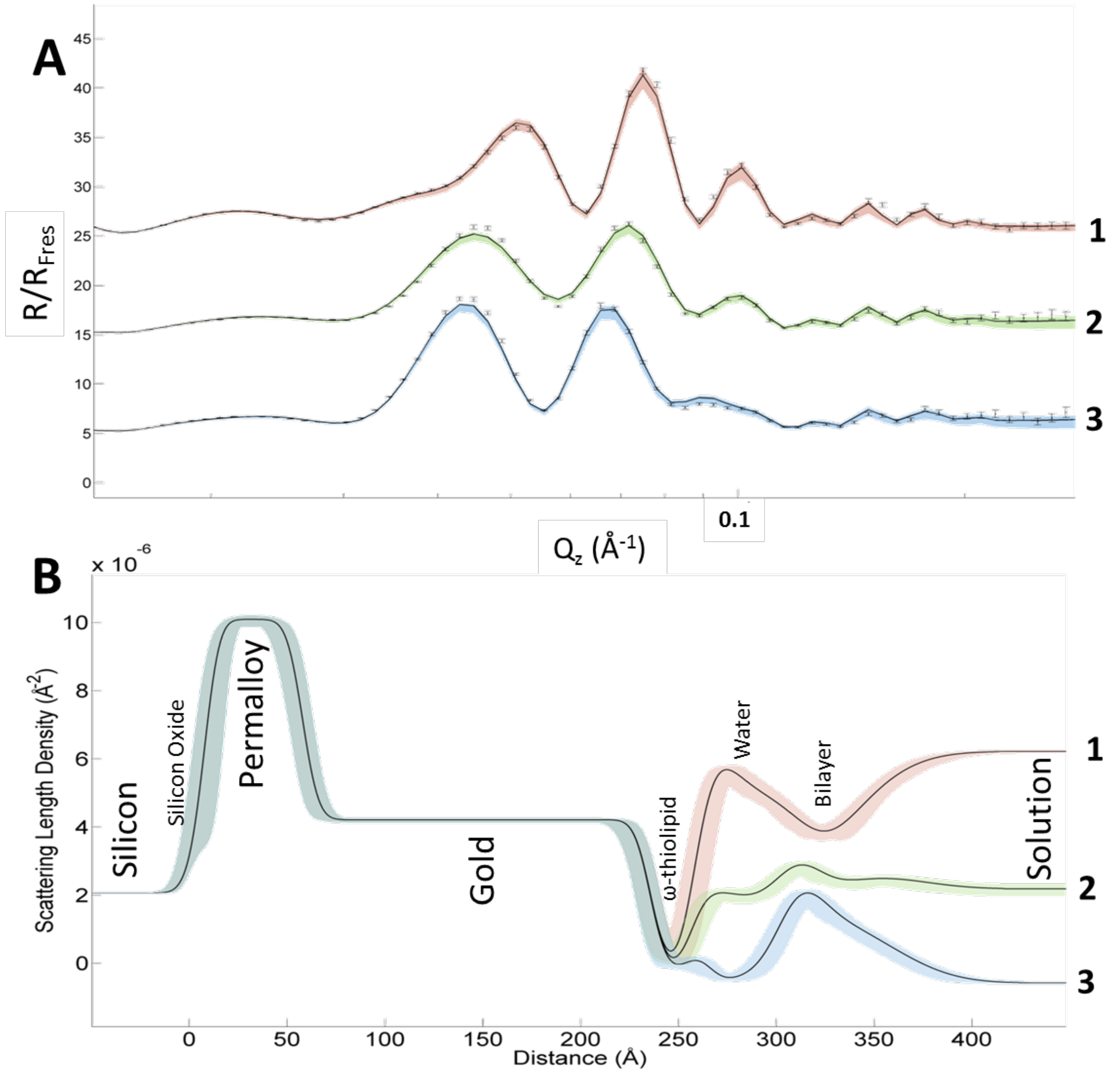

Figure S7, Neutron reflectometry profile and model data fits (A) and the scattering length density profiles these fits describe (B) for sample 4, an asymmetric d-DPPC (inner leaflet) : RaLPS (outer leaflet) bilayer deposited onto a ω-thiolipid SAM/gold/Permalloy coated silicon crystal in the presence of 20 mM HEPES pH/D 7.2 buffer with 3 mM EDTA. The three simultaneously fitted isotopic contrasts are from the sample in D_2O (1), silicon matched water (SiMW, 38% D_2O; 2) and in a H_2O (3) containing buffer subphases. Coloured shaded areas indicate the 95% confidence limits. Note how these are larger when fitting only three contrasts in the absence of two polarization sets.

Table S7, Best-fit values (maxima of the parameter probability histograms from the Bayesian analysis) obtained from the fitting of the asymmetric d-DPPC/RaLPS bilayer sample 4 in the presence of 3 mM EDTA. The bracketed ranges are the shortest 95% confidence intervals.

Parameter	Fitted Values	Priors
**Substrate Roughness (Å)**	5.74 (4.10, 7.21)	uniform (min = 4, max = 20)
**Oxide thickness (Å)**	7.98 (0.64, 14.54)	uniform (min = 0, max = 30)
**Permalloy thickness (Å)**	49.41 (48.24, 50.76)	uniform (min = 20, max = 200)
**Permalloy Roughness (Å)**	6.43 (5.30, 7.46)	uniform (min = 0.1, max = 10)
**Permalloy SLD ($Å^{-2}$)**	10.09e-06 (9.963e-06, 10.23e-06)	uniform (min = 5e-06, max = 13e-06)
**Gold Thickness (Å)**	177.47 (176.62, 178.26)	uniform (min = 150, max = 200)
**Gold Roughness (Å)**	6.48 (5.62, 7.26)	uniform (min = 5, max = 20)
**Gold SLD ($Å^{-2}$)**	4.22e-06 (4.18e-06, 4.26e-06)	uniform (min = 4e-06, max = 5e-06)
**ω-thiolipid area ($Å^2$)**	40.30 (40.01, 40.94)	uniform (min = 40, max = 100)
**ω-thiolipid head coverage (%)**	44 (29, 60)	uniform (min = 0, max = 100)
**ω-thiolipid coverage (%)**	99 (96, 100)	uniform (min = 0.5, max = 100)
**Central water thickness (Å)**	14.873 (12.73, 17.16)	gaussian (mu = 16, sigma = 1.2)
**Inner head thickness (Å)**	24.10 (21.00, 27.54)	uniform (min = 5, max = 40)
**Inner head SLD H_2O ($Å^{-2}$)**	0.43e-06 (0.01e-08, 1.54e-06)	uniform (min = 0, max = 5e-06)
**Inner head SLD D_2O ($Å^{-2}$)**	1.2864e-06 (-3.844e-07, 3.698e-06)	uniform (min = -5e-07, max = 6e-06)
**Inner head hydration (%)**	73.27 (48.91, 83.58)	uniform (min = 0, max = 100)
**Inner tails SLD ($Å^{-2}$)**	3.61e-06 (3.17e-06, 4.25e-06)	uniform (min = -4e-07, max = 8e-06)
**Inner tails thickness (Å)**	14.57 (12.43, 16.62)	gaussian (mu = 15, sigma = 1.2)
**Outer tails SLD ($Å^{-2}$)**	1.88e-06 (1.33e-06, 2.33e-06)	uniform (min = -4e-07, max = 8e-06)
**Outer tails thickness (Å)**	15.32 (13.13, 17.52)	gaussian (mu = 15, sigma = 1.2)
**Tails coverage (%)**	96 (89, 99)	uniform (min = 0, max = 100)
**Outer head SLD H_2O ($Å^{-2}$)**	2.52e-06 (1.20e-06, 3.87e-06)	uniform (min = 1e-06, max = 4e-06)
**Outer head SLD D_2O ($Å^{-2}$)**	5.42e-06 (4.44e-06, 5.97e-06)	uniform (min = -5e-07, max = 6e-06)
**Outer head thickness (Å)**	29.65 (27.35, 32.09)	uniform (min = 8, max = 40)
**Outer head hydration (%)**	49 (24, 64)	uniform (min = 0, max = 100)
**Bilayer roughness (Å)**	10.80 (7.91, 13.93)	uniform (min = 5, max = 20)
**Outer headgroup roughness (Å)**	23.96 (18.23, 29.35)	uniform (min = 5, max = 40)

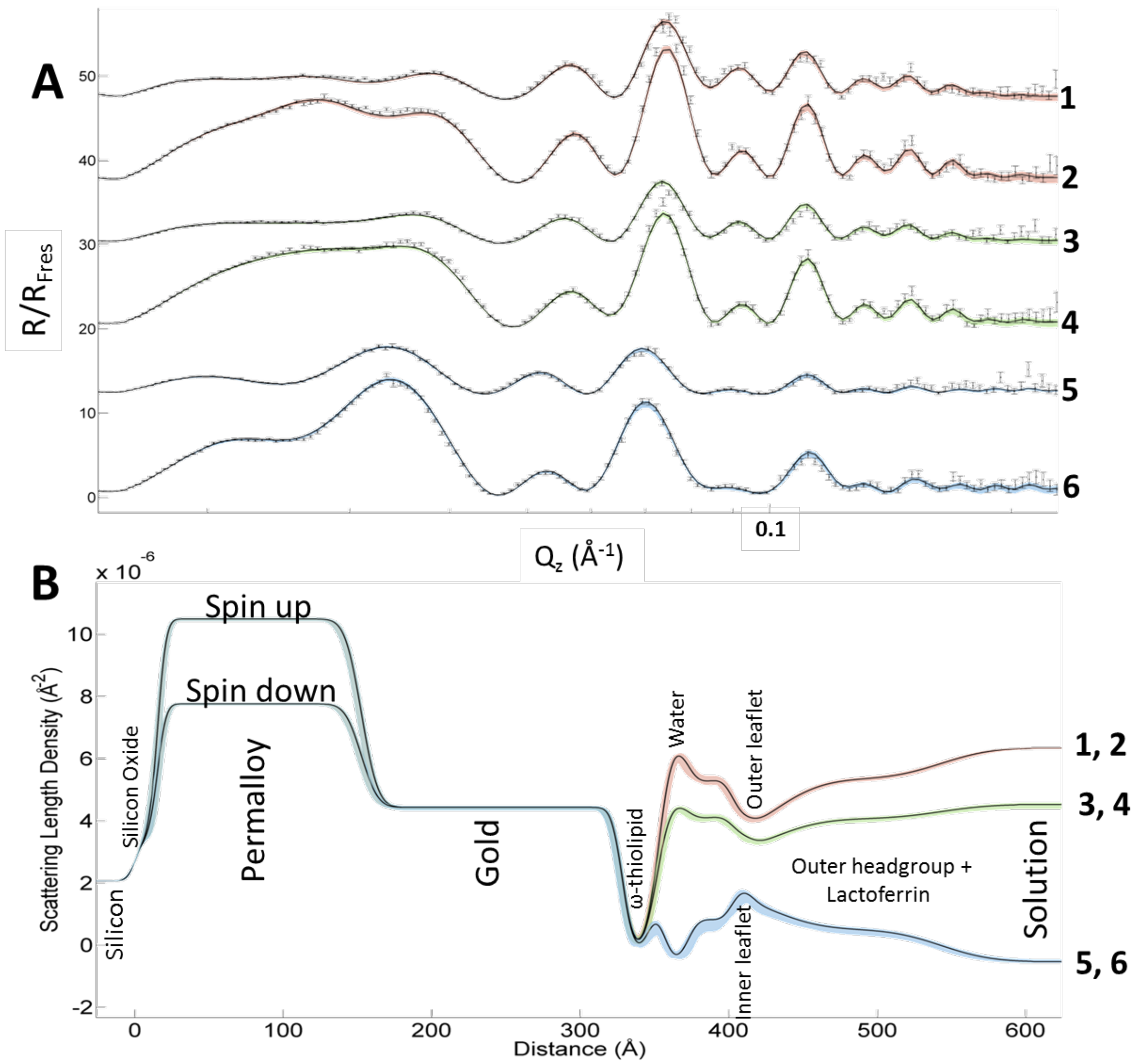

Figure S8, Neutron reflectometry profile and model data fits (A) and the scattering length density profiles these fits describe (B) for sample 3, an asymmetric d-DPPC (inner leaflet) : RaLPS (outer leaflet) bilayer deposited onto a ω-thiolipid SAM/gold/Permalloy coated silicon crystal in the presence of 0.04 mg/ml lactoferrin in 20 mM HEPES pH/D 7.2 buffer. The six simultaneously fitted isotopic and magnetic contrasts are from the sample measured in a spin down (1) and spin up (2) beam (with respect to the magnetization of the Permalloy layer) in a D_2O containing buffer subphase, spin down (3) and spin up (4) beam configuration in gold matched water (AuMW, 75% D_2O) containing subphase and spin down (5) and spin down (6) beam configuration in a H_2O containing buffer subphase. Coloured shaded areas indicate the 95% confidence limits.

Table S8, Best-fit values (maxima of the parameter probability histograms from the Bayesian analysis) obtained from the fitting of the asymmetric d-DPPC/RaLPS bilayer sample 3 in the presence of a 0.04 mg ml Lactoferrin solution. The bracketed ranges are the shortest 95% confidence intervals.

Parameter	*Fitted Values*	*Priors*
**Substrate Roughness (Å)**	4.58 (4.03, 5.49)	uniform (min = 4, max = 20)
**Oxide thickness (Å)**	14.75 (12.58, 16.50)	uniform (min = 0, max = 30)
**Permalloy thickness (Å)**	137.26 (136.86, 137.70)	uniform (min = 100, max = 200)
**Permalloy SLD spin down beam ($Å^{-2}$)**	7.76e-06 (7.74e-06, 7.79e-06)	uniform (min = 5e-06, max = 13e-06)
**Permalloy SLD spin up beam ($Å^{-2}$)**	10.49e-06 (10.46e-06, 10.53e-06)	uniform (min = 5e-06, max = 13e-06)
**Permalloy Roughness (Å)**	8.73 (8.26, 9.17)	uniform (min = 0.1, max = 10)
**Gold Thickness (Å)**	175.6 (175.13, 176.08)	uniform (min = 150, max = 200)
**Gold Roughness (Å)**	5.74 (5.24, 6.23)	uniform (min = 2, max = 20)
**Gold SLD ($Å^{-2}$)**	4.44e-06 (4.39e-06, 4.48e-06)	uniform (min = 4e-06, max = 5e-06)
**ω-thiolipid area ($Å^{2}$)**	43.674 (41.714, 45.153)	uniform (min = 40, max = 100)
**ω-thiolipid head coverage (%)**	92 (78, 100)	uniform (min = 0, max = 100)
**ω-thiolipid coverage (%)**	99(96, 100)	uniform (min = 50, max = 100)
**Central water thickness (Å)**	17.31(15.55, 19.10)	gaussian (mu = 16, sigma = 1.2)
**Inner head thickness (Å)**	30.03 (28.13, 31.86)	uniform (min = 5, max = 40)
**Inner head SLD H_2O ($Å^{-2}$)**	3.46e-06 (1.55e-06, 4.78e-06)	uniform (min = 0, max = 5e-06)
**Inner head SLD D_2O ($Å^{-2}$)**	3.0307e-06 (1.8744e-06, 4.6027e-06)	uniform (min = -5e-07, max = 6e-06)
**Inner head hydration (%)**	64 (35, 75)	uniform (min = 0, max = 100)
**Inner tails SLD ($Å^{-2}$)**	2.92e-06 (2.68e-06, 3.18e-06)	uniform (min = -4e-07, max = 8e-06)
**Inner tails thickness (Å)**	12.36 (10.33, 14.52)	gaussian (mu = 15, sigma = 1.2)
**Outer tails SLD ($Å^{-2}$)**	2.37e-06 (2.21e-06, 2.52e-06)	uniform (min = -4e-07, max = 8e-06)
**Outer tails thickness (Å)**	13.98 (11.91, 16.18)	gaussian (mu = 15, sigma = 1.2)
**Tails coverage (%)**	79 (74, 83)	uniform (min = 0, max = 1)
**Bilayer roughness (Å)**	5.82 (5.03, 7.49)	uniform (min = 5, max = 20)
**Outer headgroup / Lactoferrin layer SLD H_2O ($Å^{-2}$)**	2.63e-06 (1.89e-06, 3.52e-06)	uniform (min = -5e-07, max = 6e-06)

**Outer headgroup / Lactoferrin layer SLD D_2O (Å^{-2})**	3.33e-06 (2.37e-06, 3.98e-06)	uniform (min = 1e-06, max = 4e-06)
**Outer headgroup / Lactoferrin layer thickness (Å)**	114.21 (110.71, 117.67)	uniform (min = 8, max = 200)
**Outer head hydration (%)**	44 (13, 63)	uniform (min = 0, max = 100)
**Outer headgroup / Lactoferrin layer roughness**	26.53 (24.55, 28.63)	uniform (min = 5, max = 30)

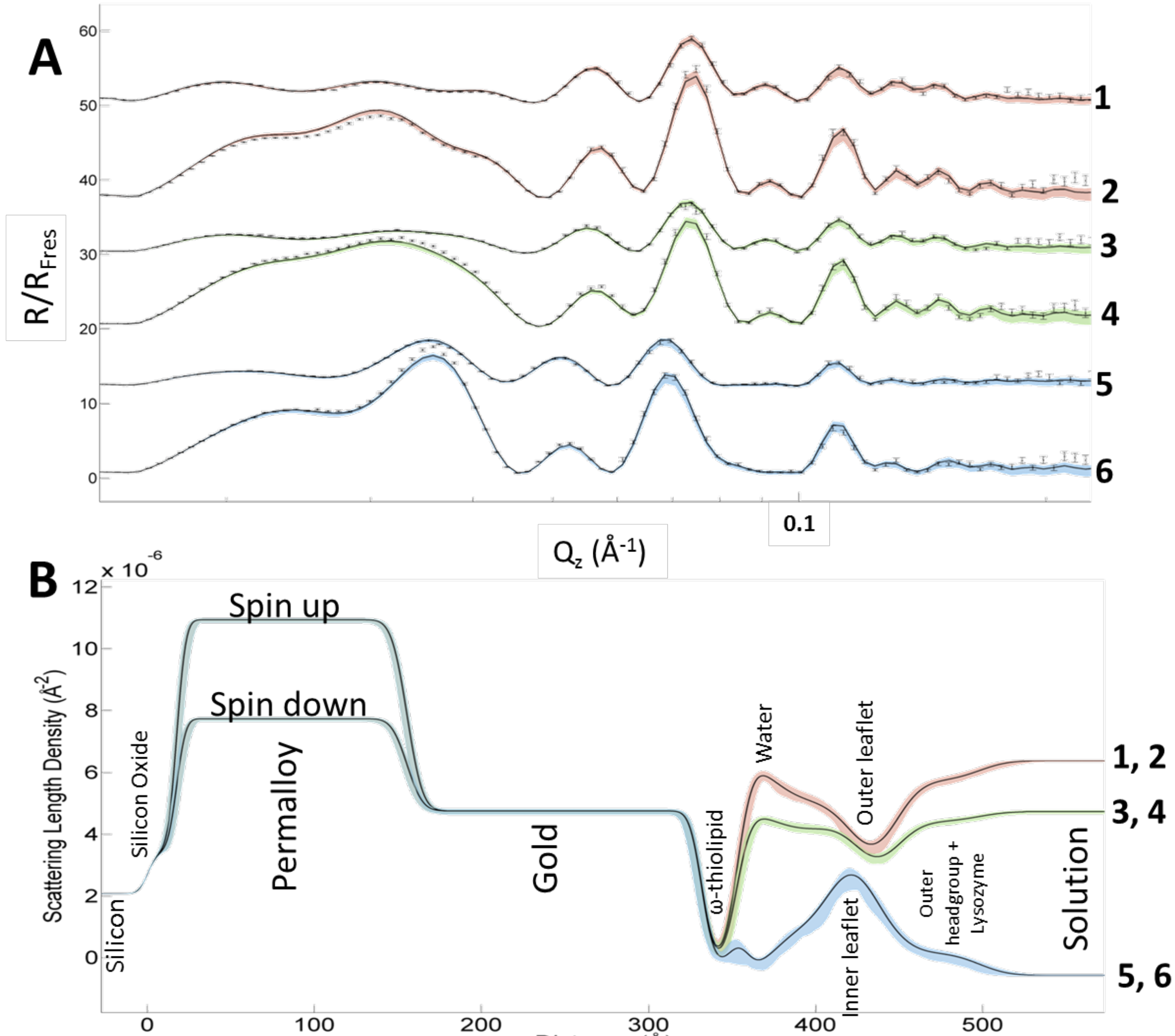

Figure S9, Neutron reflectometry profile and model data fits (A) and the scattering length density profiles these fits describe (B) for sample 1, an asymmetric d-DPPC (inner leaflet) : RaLPS (outer leaflet) bilayer deposited onto a ω-thiolipid SAM/gold/Permalloy coated silicon crystal in the presence of 0.1 mg/ml lysozyme in 20 mM HEPES pH/D 7.2 buffer. The six simultaneously fitted isotopic and magnetic contrasts are from the sample measured in a spin down (1) and spin up (2) beam (with respect to the magnetization of the Permalloy layer) in a D_2O containing buffer subphase, spin down (3) and spin up (4) beam configuration in gold matched water (AuMW, 75% D_2O) containing subphase and spin down (5) and spin down (6) beam configuration in a H_2O containing buffer subphase. Coloured shaded areas indicate the 95% confidence limits. Note how these are larger when fitting only three contrasts in the absence of two polarization sets.

Table S9, Best-fit values (maxima of the parameter probability histograms from the Bayesian analysis) obtained from the fitting of the asymmetric d-DPPC/RaLPS bilayer sample 1 in the presence of a 0.1 mg ml Lysozyme solution. The bracketed ranges are the shortest 95% confidence intervals.

Parameter	Fitted Values	Priors
**Substrate Roughness (Å)**	4.28 (4.01, 4.80)	uniform (min = 4, max = 20)
**Oxide thickness (Å)**	17.2 (15.02, 19.12)	uniform (min = 0, max = 30)
**Permalloy thickness (Å)**	137.79 (137.35, 138.29)	uniform (min = 100, max = 200)
**Permalloy SLD spin down beam ($Å^{-2}$)**	7.74e-06 (7.69e-06, 7.77e-06)	uniform (min = 5e-06, max = 1.3e-05)
**Permalloy SLD spin up beam ($Å^{-2}$)**	10.94e-06 (10.89e-06, 10.98e-06)	uniform (min = 5e-06, max = 13e-06)
**Permalloy Roughness (Å)**	7.33 (6.37, 8.21)	uniform (min = 0.1, max = 10)
**Gold Thickness (Å)**	176.15 (175.37, 176.84)	uniform (min = 150, max = 200)
**Gold Roughness (Å)**	5.71 (5.07, 6.57)	uniform (min = 5, max = 20)
**Gold SLD ($Å^{-2}$)**	4.75e-06 (4.69e-06, 4.81e-06)	uniform (min = 4e-06, max = 5e-06)
**ω-thiolipid area ($Å^2$)**	44.99 (41.15, 48.95)	uniform (min = 40, max = 100)
**ω-thiolipid head coverage (%)**	57 (25, 90)	uniform (min = 0, max = 100)
**ω-thiolipid coverage (%)**	98 (94, 100)	uniform (min = 0.5, max = 100)
**Central water thickness (Å)**	16.81 (14.87, 18.76)	gaussian (mu = 16, sigma = 1.2)
**Inner head thickness (Å)**	37.48 (34.63, 39.75)	uniform (min = 5, max = 40)
**Inner head SLD H_2O ($Å^{-2}$)**	3.38e-06 (1.52e-06, 4.93e-06)	uniform (min = 0, max = 5e-06)
**Inner head SLD D_2O ($Å^{-2}$)**	3.1226e-06 (1.3409e-06, 4.5647e-06)	uniform (min = -5e-07, max = 6e-06)
**Inner head hydration (%)**	61 (36, 75)	uniform (min = 0, max = 100)
**Inner tails SLD ($Å^{-2}$)**	4.52e-06 (3.76e-06, 5.70e-06)	uniform (min = -4e-07, max = 8e-06)
**Inner tails thickness (Å)**	15.92 (14.05, 18.09)	gaussian (mu = 15, sigma = 1.2)
**Outer tails SLD ($Å^{-2}$)**	1.78e-06 (9.65e-07, 2.3e-06)	uniform (min = -4e-07, max = 8e-06)
**Outer tails thickness (Å)**	16.07(14.12, 18.06)	gaussian (mu = 15, sigma = 1.2)
**Tails coverage (%)**	97 (91, 100)	uniform (min = 0, max = 100)
**Bilayer roughness (Å)**	12.88 (7.36, 18.22)	uniform (min = 5, max = 20)
**Outer headgroup / Lysozyme layer SLD H_2O ($Å^{-2}$)**	3.12e-06 (1.34e-06, 4.56e-06)	uniform (min = -5e-07, max = 6e-06)
**Outer headgroup / Lysozyme layer SLD D_2O ($Å^{-2}$)**	3.47e-06 (2.33e-06, 4.53e-06)	uniform (min = -5e-07, max = 6e-06)
**Outer headgroup / Lysozyme layer thickness (Å)**	53.73 (48.17, 59.85)	uniform (min = 8, max = 200)

**Outer head hydration (%)**	44 (13, 63)	uniform (min = 0, max = 100)
**Outer headgroup / Lysozyme layer roughness**	26.53 (24.55, 28.63)	uniform (min = 5, max = 30)

1. Hughes, A.V., et al., *High Coverage Fluid-Phase Floating Lipid Bilayers supported by Ω-Thiolipid SAMs.* J. Roy. Soc. Interface, 2014. **11** (98): p. 20140447.
2. Clifton, L.A., et al., *Asymmetric phospholipid: lipopolysaccharide bilayers; a Gram-negative bacterial outer membrane mimic.* J. Roy. Soc. Interface, 2013. **10**(89): p. 20130810-20130810.
3. Hughes, A.V., et al., *Floating lipid bilayers deposited on chemically grafted phosphatidylcholine surfaces.* Langmuir, 2008. **24**(5): p. 1989-1999.
4. Clifton, L.A., C. Neylon, and J.H. Lakey, *Examining protein-lipid complexes using neutron scattering.* Methods in molecular biology (Clifton, N.J.), 2013. **974**: p. 119-50.
5. Penfold, J., R. Ward, and W. Williams, *A time-of-flight neutron reflectometer for surface and interfacial studies.* J. Phys. E: Sci. Instrum, 1987. **20**: p. 1411-1417.
6. Williams, W., *Polarized Neutrons*. 1988: Oxford University Press. 98-162.
7. Alsnielsen, J., et al., *Principles And Applications Of Grazing-incidence X-ray And Neutron-scattering From Ordered Molecular Monolayers At The Air-water-interface.* Physics Reports-Review Section of Physics Letters, 1994. **246**(5): p. 252-313.
8. Holt, S.A., et al., *An ion channel containing model membrane: structural determination by magnetic contrast neutron reflectometry* Soft Matter, 2009. **5** p. 2576 - 2586.
9. Le Brun, A.P., et al., *Monitoring the assembly of antibody-binding membrane protein arrays using polarised neutron reflection.* European Biophysics Journal with Biophysics Letters, 2008. **37**(5): p. 639-645.
10. Hughes, A.V., *Rascal*. 2013: Sourceforge.
11. Parratt, L.G., *Surface Studies of Solids by Total Reflection of X-Rays.* Physical Review, 1954. **95**(1): p. 359-369.
12. Armen, R.S., O.D. Uitto, and S.E. Feller, *Phospholipid component volumes: Determination and application to bilayer structure calculations.* Biophysical Journal, 1998. **75**(2): p. 734-744.
13. Clifton, L.A., et al., *Effect of divalent cation removal on the structure of gram-negative bacterial outer membrane models.* Langmuir, 2015. **31**(1): p. 404-12.
14. Sivia, D.S. and J.R.P. Webster, *The Bayesian approach to reflectivity data.* Physica B, 1998. **248**: p. 327-337.
15. Sivia, D.S. and J. Skilling, *Data Analysis - A Bayesian Tutorial*. Second Edition ed. 2006: Oxford University Press
16. Haario, H., E. Saksman, and J. Tamminen, *An adaptive Metropolis algorithm.* Bernoulli, 2001. **7**(2): p. 223-242.
17. Haario, H., et al., *DRAM: Efficient adaptive MCMC.* Statistics and Computing, 2006. **16**(4): p. 339-354.
18. Piggot, T.J., A. Pineiro, and S. Khalid, *Molecular Dynamics Simulations of Phosphatidylcholine Membranes: A Comparative Force Field Study.* Journal of Chemical Theory and Computation, 2012. **8**(11): p. 4593-4609.
19. Berendsen, H.J.C., D. van der Spoel, and R. van Drunen, *GROMACS - A Message-passing Parallel Molecular-dynamics Implementation.* Computer Physics Communications, 1995. **91**(1-3): p. 43-56.
20. Van der Spoel, D., et al., *GROMACS: Fast, flexible, and free.* Journal of Computational Chemistry, 2005. **26**(16): p. 1701-1718.
21. Hess, B., et al., *GROMACS 4: Algorithms for highly efficient, load-balanced, and scalable molecular simulation.* Journal of Chemical Theory and Computation, 2008. **4**(3): p. 435-447.

22. Oostenbrink, C., et al., *A biomolecular force field based on the free enthalpy of hydration and solvation: The GROMOS force-field parameter sets 53A5 and 53A6.* Journal of Computational Chemistry, 2004. **25**(13): p. 1656-1676.
23. Berendsen, H.J.C., et al., *Interaction Models for Water in Relation to Protein Hydration.* , in *Intermolecular Forces*, B. Pullman, Editor. 1981, D. Reidel Publishing Company. p. 331-342.
24. Nose, S., *A Molecular-dynamics Method For Simulations In The Canonical Ensemble.* Molecular Physics, 1984. **52**(2): p. 255-268.
25. Hoover, W.G., *Canonical Dynamics - Equilibrium Phase-space Distributions.* Physical Review A, 1985. **31**(3): p. 1695-1697.
26. Parrinello, M. and A. Rahman, *Polymorphic Transitions In Single-crystals - A New Molecular-dynamics Method.* Journal of Applied Physics, 1981. **52**(12): p. 7182-7190.
27. Nose, S. and M.L. Klein, *Constant Pressure Molecular-dynamics For Molecular-systems.* Molecular Physics, 1983. **50**(5): p. 1055-1076.
28. Essmann, U., et al., *A Smooth Particle Mesh Ewald Method.* Journal of Chemical Physics, 1995. **103**(19): p. 8577-8593.
29. Hess, B., *P-LINCS: A parallel linear constraint solver for molecular simulation.* Journal of Chemical Theory and Computation, 2008. **4**(1): p. 116-122.
